# Prospective Evaluation of Children with Robin Sequence following Tübingen Palatal Plate Therapy

**DOI:** 10.3390/jcm12020448

**Published:** 2023-01-05

**Authors:** Josephine Effert, Simone Uhlig, Cornelia Wiechers, Mirja Quante, Christian F. Poets, Matthias C. Schulz, Siegmar Reinert, Michael Krimmel, Bernd Koos, Christina Weise

**Affiliations:** 1 Department of Orthodontics, University Hospital Tuebingen, Osianderstr. 2-8, 72076 Tuebingen, Germany; 2 Department of Neonatology, University Hospital Tuebingen, Calwerstr. 7, 72076 Tuebingen, Germany; 3 Department of Oral and Maxillofacial Surgery, University Hospital Tuebingen, Osianderstr. 2-8, 72076 Tuebingen, Germany

**Keywords:** cleft palate, craniofacial disorder, upper airway obstruction, growth, orthodontic treatment, mandibular retrognathia, functional orthodontic parameters, jaw index, non-invasive treatment, personalized medical appliance

## Abstract

Background: To assess the long-term functional orthodontic outcome of the Tübingen palatal plate (TPP) in children with Robin sequence (RS) in comparison to age- and sex-matched healthy controls. Methods: Between 09/2019 and 10/2020, we performed orthodontic assessments in 41 children at our Department of Orthodontics. Included were patients with RS (17 non-syndromic; four syndromic) and healthy controls (*n* = 22, average age in both groups 9.9 y). Facial analyses of 2D images, digital study casts and cephalometric measurements were made. Results: The orthodontic examinations showed no statistically significant group differences regarding functional extraoral, intraoral and pharyngeal parameters, or in skeletal patterns. The relationship between the upper and lower incisors was significantly increased (overjet 4 (2–10) vs. 3 (0–9) mm; *p* = 0.01) with a significant deficit in the lower face proportions (Jaw Index 4.15 (1.9–9.6) vs. 2.98 (0–9); *p* = 0.02; Facial convexity angle 157 (149–173) vs. 159 (149–170); *p* = 0.01). Conclusion: Children with RS treated with the TPP showed normal long-term functional orthodontic outcomes, thanks to the functional adaption of the stomatognathic system. However, soft tissue growth did not completely match skeletal growth, resulting in a more convex facial profile.

## 1. Introduction

**Figure 1:**
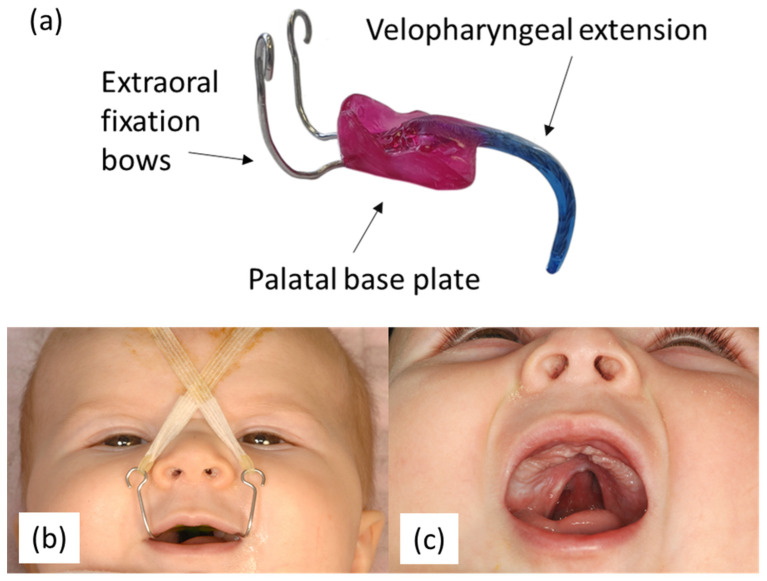
Tübingen palatal plate (TPP) orthodontic appliance: (**a**) construction of the TPP bases on two extraoral fixation bows, a velopharyngeal extension (blue) and a palatal base plate (pink); (**b**) Robin sequence patient with inserted TPP and its fixation by extraoral tapes; (**c**) Robin sequence patient without the inserted TPP showing the cleft palate.

Robin sequence (RS) is a rare congenital craniofacial anomaly, first described by the stomatologist Pierre Robin in 1923 [1], which involves the triad of mandibular retrognathia, glossoptosis and resultant upper airway obstruction (UAO). In addition, 80–90% of affected patients also have a U-shaped cleft palate [1]. The prevalence of RS in Europe is 12:100,000 live births [2,3,4,5] and it can occur as an isolated anomaly or in combination with a syndrome. Patients with RS suffer from severe functional difficulties, such as feeding problems and failure to thrive; therefore, treatment from early after birth is necessary. The treatment relies on an interdisciplinary approach performed by neonatologists, cranio-maxillofacial surgeons, ear, nose and throat specialists, speech therapists, orthodontists and psychologists, which will extend until the patient’s adolescence and beyond [6]. Its duration depends on the severity of the given craniofacial malformation. One of the first problems to tackle in these patients is UAO. For this, different treatment concepts are used world-wide, ranging from prone positioning in mild cases, to a nasopharyngeal airway or continuous positive airway pressure in moderate to severe ones. Invasive surgical procedures include tongue lip adhesion, mandibular distraction or tracheostomy [6,7,8]. Among the non-invasive approaches, a treatment concept centered around the Tübingen palatal plate (TPP) is comparatively well studied [9,10,11,12,13,14,15]. The TPP is an individually manufactured orthodontic appliance (Figure 1), which consists of a palatal base plate, a velopharyngeal extension and two extraoral fixation bows. Construction parameters of the velopharyngeal extension are patient individualized, to assume its optimal position just above the epiglottis. Moreover, the design needs to be suitable to push the base of the tongue forward, opening the airways effectively while avoiding high pressure on the base of the tongue. A successful TPP is able to improve respiration and the related oxygen saturation, as well as promote a correct physiological swallowing. In addition, extraoral fixation tapes are necessary to be attached to the extraoral bows to counterbalance the pressure that the tongue exerts on the velopharyngeal extension.

**Figure 2:**
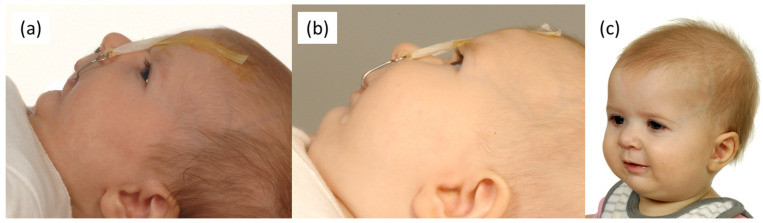
Mandibular growth development of a patient with Robin sequence treated with the Tübingen palatal plate (TPP) directly after birth: (**a**) facial profile after two months of TPP treatment; (**b**) facial profile after four months of TPP treatment; (**c**) patient after successful TPP treatment with eight months.

The indication for TPP treatment is given by the extent of UAO, the combination of clinical parameters, like breathing sounds, as well as orthodontic intra- and extraoral examinations. To evaluate the severity of obstruction, an overnight sleep study is performed [9]. The aim is to start TPP treatment as early as possible after birth, which takes place in the first six to nine months (Figure 2). Due to the physiological growth of the patient it needs to be renewed every three to four months. For this purpose, an intraoral scan and semi-digital workflow approach is the current TPP manufacturing approach [16,17,18]. The TPP treatment alongside feeding training not only resolves the UAO, but also results in a normal weight gain with bottle feeding. This is believed to induce mandibular growth based on a functional adaption, following the paradigm of “form follows function”.

Due to the craniofacial malformation underlying RS, special dentoskeletal patterns in growth and development occur. To achieve functional, harmonious and aesthetic results, these special growth patterns have to be considered by the treating orthodontist. In the current literature, the presence of mandibular catch-up growth is controversially discussed in patients with RS [19]. The catch-up growth concept implies the potential of the mandible in patients with RS to grow faster than in healthy infants, and to resolve the maxillo-mandibular discrepancy [20,21,22]. Most studies argue that catch-up growth is not always sufficient to reach normal dimensions [23,24,25,26,27,28,29]. Only few objective studies proved the opposite [30,31,32]. To evaluate growth, functional and dentofacial patterns, a clinical examination that is both comprehensive and systematic is fundamental prior to and during orthodontic treatment. This includes examining facial proportions and dental dimensions, such as malocclusion, dental alignment, skeletal growth patterns, deleterious habits, as well as speech and the function of the temporomandibular joint. For these purposes, 2D images, dental records, such as orthodontic study models, and cephalometric radiographs can be used. These images and radiographs can be assessed with defined landmarks to construct angles and lines, which are compared to reference values. These assessments are standardized and defined according to different orthodontic evaluations. No previous study has yet examined the described dentofacial orthodontic patterns in patients with RS who were treated with the TPP early after birth.

The aim of this study was to evaluate the long-term dentofacial growth of school-aged patients with RS treated with a TPP as newborns compared to age- and sex-matched healthy controls. Thereby, our aim was to investigate the long-term functional treatment results of the TPP therapy. We hypothesized that children with RS show worse results in stomatognathic function and their aesthetic facial soft-tissue profile, compared to healthy children.

## 2. Materials and Methods

### 2.1. Study Design

The study was designed as a prospective, explorative and cross-sectional case-control study over a period of 13 months at our interdisciplinary Centre for Cleft Lip Palate and Craniofacial Malformation at the University Hospital Tübingen. Data collection took place in collaboration with the Department of Neonatology, in order to investigate the long-term outcome of the TPP therapy regarding comprehensive orthodontic history and diagnostic surveys in patients with RS at school age, compared to age- and sex-matched healthy controls. An informed consent form for study participation was signed by legal guardians. We contacted the parents of patients with RS by mail and/or telephone. Age- and sex-matched controls were recruited via word of mouth and a mailing list of Tübingen University and its Hospital. All orthodontic examinations were non-invasive, non-stressful for participants and could be performed in one session of approximately 60 min. The study was approved by the institutional ethics committee of Tübingen University (approval number: 361/2019BO1, date of approval: 1 April 2019) and registered with “Deutsches Register Klinische Studien” (trial number DRKS00017770).

### 2.2. Participants

Two cohorts were invited to participate. Children with RS were admitted to our intermediate neonatal care unit between 01/2008 and 12/2012 and successfully treated with the TPP therapy concept during infancy. Patients with RS were diagnosed directly after birth based on mandibular retrognathia, respiratory distress and overnight polygraphy, which yielded an obstructive apnea-index (OAI) of >3/h. TPP therapy took place in the first six to nine months after birth. The control group consisted of 21 healthy age- (+/− 6 months) and sex-matched children.

The following inclusion criteria were applied:

(1) Group of children with RS: successful TPP therapy initiated after birth;
(2) Control group: no congenital malformation or chromosomal aberration;
(3) Born between 1 January 2008 and 31 December 2012.

Exclusion criteria were defined as follows:

(1) Missing parental consent or participant’s assent;
(2) Group of children with RS: not treated with the TPP or receiving additional invasive treatments (i.e., tracheostomy);
(3) Control group: craniofacial malformation, obstructive sleep apnea and other syndromes.

### 2.3. Instruments

To assess skeletodental patterns, an orthodontic examination was performed according to the parameters shown in Table 1. The extraoral inspection included a functional assessment of the perioral muscle and an evaluation of body position, as well as the existence of deleterious habits. The intraoral evaluation included the stage of dentition, the Angle malocclusion classification of the left and right side [33], the relationship of the front teeth in sagittal and vertical dimension (overjet and overbite), the dental midline shift of the upper and lower jaws, and the shift of the whole lower jaw in a resting position. Furthermore, the region of the pharynx and the tongue were examined according to the type of phonation, e.g., interdental or nasal; the size of the tonsils were documented and the tongue position and the swallowing pattern were evaluated, e.g., somatic or visceral. For facial proportion analysis, biomedical 2D lateral and frontal facial images were made by a digital single lens reflector camera Canon EOS 450D with a ring flash (Canon Germany GmbH, Krefeld, Germany). For the lateral profile view the camera was placed parallel to the Frankfurt’s horizontal plane. The child was asked to sit upright, turn the face towards the right side, looking straight ahead so that the tip of opposite eyelash was visible and the facial muscles were in a relaxed position [34]. For taking the front facial image, the patient was turned to face the camera straight, eyes open, with the ears exposed, looking directly into the camera, while the tip of the nose was the center of the frame [34].

Intraoral diagnostic records were taken using a 3D intraoral scan (Trios 3^®^ 3Shape, Copenhagen, Denmark) of the maxilla and mandible, including bite registration in order to manufacture a digital cast model. Digital study models were assessed to evaluate symmetry and space deficiency or excess within the dental arches. The size of each tooth, the arch width and length were measured to assess the effective available space, as these factors have considerable implications in orthodontic treatment planning. For this purpose, the analyses of Moyers and Harth were used [39,40,43].

We requested lateral cephalometric radiographs from the treating orthodontists or dentists to determine growth trend patterns and to diagnose the severity of the malocclusion, if available and after obtaining consent. The lateral radiographs showed the skeletal sagittal relationship of the jaws, the growth trend of the mandibular and maxillary plane in relation to the skull base, the inclination and position of the upper and lower incisors as well as the posterior airway space (PAS) [41,44]. These cephalometric analyses involved the location of specific landmarks, which are used to measure linear and angular variables reflecting the relationship of the anatomical growth of each patient. In this study, we used the analysis method of Hasund to evaluate anatomical growth patterns [42].

The Jaw Index was determined according to van Haven et al. [35]. Three different values are required for this purpose. The maxillary arch was measured subnasally from the left to the right tragus and the mandibular arch via the pogonion from the left to the right tragus, in millimeters with a measuring tape. The overbite was evaluated during the orthodontic examination and defined as the vertical overlap of the maxillary in relation to the mandibular incisor and measured in millimeters. Higher index values were obtained with a smaller mandible and an increased overjet. All evaluations of the digital models and the lateral radiographs were made with the imaging software OnyxCeph^3TM^ (Image Instruments GmbH, Chemnitz, Germany).

### 2.4. Statistical Data Analysis

Patient data were collected and pseudonymized in an Excel^®^ sheet (Microsoft Inc., Redmond, Washington, USA). Statistical evaluation, descriptive statistics and analyses were performed using JMP (Version 15.2.0, SAS Institute Inc., Cary, USA). Shapiro-Wilk test was used as a test for normality. Differences between groups were analyzed for normally distributed data with a t-test and for skewed data with a Wilcoxon Sign Rank test. Pearson’s χ^2^ statistics were used to examine differences between non-continuous variables. Statistical significance was considered at a *p*-value of < 0.05.

## 3. Results

### 3.1. Characteristics of the Study Participants

Of 93 children with isolated or syndromic RS treated with the TPP in our center, 69 fulfilled inclusion criteria; 18 could not be reached and 30 could not be included (restrictions due to the COVID-19 pandemic, long distance to travel, nonappearance on the day of examination, no interest in the study), yielding a final study population of 21 patients with RS. All patients presented with a cleft palate, four (19%) were syndromic patients with RS (Stickler syndrome (2), Cat-eye syndrome, 4q deletion). We examined a total of 43 participants. The mean age at the day of examination was 9.9 years (range 7.8–12.1) in patients with RS and 9.9 years (range 7.6–12.1) in controls, respectively. The sex ratio was 12 males, nine females in the group of children with RS and 11 males to 10 females in controls.

### 3.2. Orthodontic History

Table 2 presents the results of the orthodontic evaluation comparing children with RS against healthy controls. There was a tendency towards more muscle activity in children with RS (35.3 %) compared to the control group (10.5 %). Regarding the position of the lip, all children in the control group had a competent lip closure, whereas 14.3% of children with RS had incompetent lip closure. In both groups the body posture was balanced. Children with RS had less habits like thumb sucking. Most patients with RS had a mixed late stage of dentition. Compared to the healthy control group, the stage of dentition was slightly earlier. Regarding the malocclusion according to Angle, the results of both groups were balanced with most children having a common Angle class II malocclusion. However, children with RS exhibited more Angle class III malocclusion. In 40.5% of patients with RS had a dentoalveolar upper matching midline of the face, versus 46.0% in the control group. Regarding the dental midline in the lower jaw, in 18.9% of patients with RS, they had a matching line of the face compared to 27% of the healthy ones. A higher proportion of patients with RS had a mandibular deviation by ≤5 mm (14.3% versus 10.5%). In all, 14.3% of children with RS had an interdental phoniation, which was rarely seen in controls, while patients with RS had smaller tonsils. No significant differences in swallowing patterns were identified in both groups. With respect to the tongue position, children with RS showed more often regular positioning than the healthy control group. The fast-check examination of the temporomandibular joint was significantly more often conspicuous in patients with RS compared to controls (31.6% versus 0.0%). In all, 13.3% of children with RS were snoring; in the healthy control group none reported this. The relationship of the front teeth was significantly different between groups in overjet (4 mm versus 3 mm) as well as in overbite (4.3 mm versus 3 mm.). The mouth opening distance measured at the incisal edge was significantly lower in children with RS (36 mm versus 44 mm), whereas the Jaw Index was significantly higher in patients with RS (4.2 versus 3).

### 3.3. Facial Soft Tissue Analysis of 2D Images

The results of the facial soft tissue analysis showed that patients with RS had a significantly more pronounced convex facial profile, i.e., mandibular retrognathia and a deficit in the lower face proportions, compared to control group children as evident by several angle values (Table 3). The profile and the facial convexity angle according to the analysis of A.M. Schwarz (SNPog’-Pn, Gl’-Sn-Pog’) showed a significantly higher median value in children with RS. Moreover, these patients had a significantly lower Z-Angle according to Merrifield, with a median value of 62.1° compared to 71.3° in controls. The nasolabial angles (Cotg-Sn-Ls) of both groups were balanced (112.6° versus 114.5°), but higher than the reference value of 106.5°. The angle of the lower face (Sn-Tra-Me’) differed significantly between groups (39.6° versus 35.8°). The angle of the midface (N’-Tra-Sn) was significantly lower in children with RS (24.1° versus 25.5°). The angle of the chin (GoL-f-Me-GoR) was increased to 111 % compared to the reference value of 66%. The relations between higher/lower face and mid/lower face were balanced in both groups.

### 3.4. Digital Casts Analysis

Table 4 shows the analyses of the dental digital casts. The tooth space in the respective dental arches of both groups was balanced. The largest difference between both groups occurred regarding the Tonn Index of the sum of the widths of the upper incisors from the sum of the widths of the lower incisors, which was 72.5 % in RS and 76.0 % in controls. The tooth arch width analysis according to Harth showed a significantly larger posterior arch width of the upper jaw (PAWUJ) in the group of children with RS (48 mm versus 46 mm, *p* = 0.01).

### 3.5. Skeletal Cephalometric Analysis

Eleven children with RS and six controls provided lateral cephalometric radiographs through their treating orthodontist or dentist. Due to the small sample size, only descriptive data are presented in Table 5. The PAS values of both groups showed the same results, where only the space in the maxillary plane was slightly smaller in children with RS than in controls (12.5 mm versus 17.05 mm). Regarding the results of the Hasund analysis, no group differences in the skeletal and dental relationship values were shown. The mandibula angle (Ar-Go-Gn) was increased in children with RS (131° versus 126°). The interincisal angle with a reference value of 110° was increased to 144° in RS. The cephalometric analysis showed that the length of the mandibula (Go-Pog’) was shortened in patients with RS (69 mm) compared to controls (77 mm).

## 4. Discussion

The aim of this study was to evaluate the orthodontic long-term functional outcome of patients with RS following TPP treatment in comparison to age- and sex-matched healthy controls. Novelties include the long-term orthodontic follow-up and the comparison with age- and sex-matched healthy controls. The results of this study show that long-term orthodontic parameters in patients with RS did not differ from those found in healthy controls. This was particularly true for functional parameters, the size of the dental arch, as well as the vertical and sagittal cephalometric values. These results confirm that the TPP is an effective, yet only minimally invasive therapy regarding its long-term treatment results. Despite this, the aesthetic facial analysis showed a significantly increased convex profile with a deficit in the lower face proportions and a tendency to an incompetent lip closure in patients with RS. This finding is in line with the current literature. For example, Ozawa et al. evaluated the facial profile of 60 isolated patients with RS and 23 controls aged 5 to 10 years [45]. They stated that due to a lack of the anterior mandibular projection in patients with RS, the convexity of the profile is usually extended. This was also confirmed by the significantly increased angles of the lower and midface of patients with RS in this study.

Cephalometric measurements showed that values for the vertical and sagittal dimension, in relation to the base of the skull, did not differ widely from those in the control group. Patients with RS have a bimaxillary retrognathia with steep mandibular planes, a narrow airway as well as bifrontal lingually tipped and crowded incisors. The gonial angle is enlarged with a shorter ramus, maxillar and mandibular length [23,26,30,46,47]. Compared to patients treated with the TPP, the length of the ramus did not differ from controls, with a minimally increased vertical growth and significantly shorter mandibula and maxilla in combination with a retrognathic face type. Regarding the functional values of the PAS, Laitinen et al. revealed a shorter space in patients with RS when performing lateral cephalograms in 35 patients with RS at a mean age of 9.5 years and again 4.3 years later [47]. This finding is in contrast to the results of this study, as patients with RS treated with the TPP had a similar PAS. This suggests that the TPP has a functional influence regarding the opening of the airway and inducing vertical and sagittal growth. Concerning the mechanical effects of the TPP, the spur location starts in the area of the soft palate and extends until just above the epiglottis, to push the tongue anteriorly, while relocating the mandible and opening the hypopharynx. In our study, the PAS was found to be enlarged exactly in this area. Therefore, TPP treatment proved to be effective not only for opening the upper airway during treatment, but also in the long-term. Moreover, the TPP is an orthodontic functional appliance inducing a stimulus for the mandibular and temporomandibular joint growth, which is based on a functional adaption and that provides an anterior growth incentive [48,49]. In combination with manual orofacial functional therapy, according to the Castillo Morales concept and intensive feeding training, the orofacial functions and interactions of the newborn were supported [50]. These assumptions were confirmed in the current study. Patients with RS had the same functional patterns as healthy individuals, such as muscle activity, mouth breathing and tongue position, which have an important influence on facial growth. This can be due to the fact that patients with RS received an intensive speech therapy. While these functional patterns can also influence positively the breathing disorder and the development of UAO, it is of greater importance that RS patients treated with the TPP have a functional balance to prevent UAO also at an older age. Furthermore, the intraoral assessment showed even better results in patients with RS. For example, they showed a higher percentage in angle class III malocclusion, which indicates that the dentoalveolar complex of the mandibula is more anterior than the maxilla.

Paladini et al. and Mermans et al. showed that the Jaw Index is a reliable instrument to assess the objective size of the lower jaw in newborns [51,52]. In this study, patients with RS showed a significantly increased index connected with a smaller mandibula and an increased overjet. This could be explained by the fact that RS patients have mandibular and maxillary retrognathic patterns and thus, the relationship of the front teeth in the sagittal dimension is significantly increased as well. This parameter has a decisive influence in the calculation of the Jaw Index. This is the first study using this index in older children, and therefore, data comparison with literature referring to newborns cannot be easily performed [35,53,54]. Nonetheless, the evaluation and use of the Jaw Index for the given application is believed to be a reliable tool to describe the relationship between mandibula and maxilla, even in older patients [14].

Furthermore, the current study contributes to the controversial discussion concerning the presence of mandibular catch-up growth in patients with RS. Regarding the results of the Jaw Index, the relationship of the front teeth and aesthetic parameters, like the convex soft-tissue profile and the tendency to an incompetent lip closure, this point is not proven. However, the children with RS in this study showed the same important functional patterns like normal PAS, tooth arch and denotalveolar dimensions. Pruzansky et al. supported this concept and its positive effects on the facial profile and respiratory system [21,22]. Hotz and Gnoiski also revealed that there was no difference in the mandibular length between patients with RS and those with an isolated cleft palate [20]. Most studies found no catch-up growth, as evident in a persistent mandibular retrognathia and convex facial profile [23,26,27,46,53,55].

As different treatment approaches for patients with RS exist, not only treatment effectiveness, but also the burden or impact on these vulnerable patients and their families must be considered. While invasive surgical methods, such as mandibular distraction osteogenesis, are efficient in relieving the UAO and achieving the desired facial profile, it may also lead to negative side effects, like nerve lesions, scar formation and damage to tooth germs that might have an impact on long-term mandibular growth [56,57]. There are no studies yet on the impact of mandibular surgery on long-term mandibular growth in patients with RS. Moreover, the fact that the TPP therapy allows to promote comparable functional parameters to healthy children, with comparatively limited burden on family and patients alike, and that no severe side effects of this therapy have been encountered yet over 25 years and >500 patients, are arguments for make it the therapy of choice for RS in newborns and small children. For these reasons, patients with RS were compared to healthy individuals, as the end goal is to achieve the function that a patient without any pathology can expect.

It is important to recognize some limitations of the study. The literature mentions two growth spurts: the so called “juvenile acceleration” at about 8 years, and the adolescent spurt, which occurs one to two years after the juvenile one [58]. The patients in this study had an average age of 9.9 years, which was between these two time periods and thus, still before the adolescent growth spurt. Especially, in this time period, part of the functional orthodontic therapy is still performed and thus, further improvement and growth of the affected jaw is expected to be achieved. Therefore, a longer follow-up would be desirable in the future, to evaluate the growth of the patients with RS during the different growth stages. Further limitations include that 26% of patients with RS meeting the study criteria could not be contacted, and that the current was a single-center study. Only 41% of patients with RS whom we contacted took part in this study, possibly due to the long distances to our center and further restrictions during the COVID-19 pandemic. Moreover, to avoid the risk of unnecessary radiation exposure, we only evaluated the X-rays of patients who had received it as part of their usual orthodontic therapy. This leads to a small sample size for children with a radiographic assessment (*n* = 11 children with RS; *n* = six healthy children), that was too low to perform reliable statistical analysis. These limitations, however, are outweighed by the knowledge gathered, as this is the first study on the orthodontic long-term functional treatment outcome of children with RS receiving TPP treatment shortly after birth, as well as its comparison to an age- and sex-matched healthy control group.

## 5. Conclusions

In conclusion, the functional and dentoalveolar parameters in school-aged children with RS after TTP treatment were comparable to those observed in healthy controls. Moreover, the vertical and sagittal skeletal values were within the normal range. Most importantly, when it comes to the PAS values, which can be an indicator for the occurrence of UAO, we confirmed that the upper airway was open in children with RS. While the dentoskeletal growth showed a positive development in children with RS, soft tissue did not completely follow its lead and could not completely mask the presence of RS, as it showed a more convex profile with a deficit in the lower face proportions. This leads to the hypothesis that TPP treatment induces a stimulus for the mandibular and temporomandibular joint growth, thanks to the functional adaption of the stomatognathic system. This not only affects the anterior growth of the mandibula after birth, but also in the long-term.

## Figures and Tables

**Table 1:** Orthodontic examinations.

1	Orthodontic anamnesis examination	- Mentalis muscle activity - Position of the lip - Body posture - Deleterious oral habits - Stage of dentition - Angle malocclusion classification [33] - Dental midline shift of the upper and lower jaw - Overjet/Overbite - Phonation - Airway examination of the tonsils - Swallowing patterns - Tongue position - Maximal possible mouth opening movement - Temporomandibular joint short check
2	Jaw Index [35]	- Using overjet values of the orthodontic examination - Alveolar overjet X maxillary/mandibular arch in millimeters
3	2D facial profile analysis with photostat lateral images	- Profile angle according to A.M. Schwarz (SNPog’-Pn) [36,37,38] - Facial convexity angle according to A.M. Schwarz (Gl’-Sn-Pog’) [36,37,38] - Z-Angle according to Merrifield - Nasolabial angle (Cotg-Sn-Ls) - Angle of the lower face (Sn-Tra-Me’) - Angle of the midface (N’-Tra-Sn) - Index of the relation between upper face and lower face (Tri-NR/NL:NR/NL-f-Me) - Index of the relation between mid-face and lowerface (f-Gl-Sub: Sub-f-Me) - Index of the relation between length and width of the face (TH-F-Me: WR-WL) - Chin angle (GoL-f-Me-GoR)
4	Dental digital casts analysis	- Space analysis in mixed dentition using Moyer’s method [39] - Analysis of the anterior and posterior arch width according to Harth’s method [40]
5	Cephalometric measurements	- Posterior airway space measured at the maxillary, mandibular and occlusal plane [41] - Cephalometric measurements according to Hasund analysis [42] - Soft tissue analysis according to A.M. Schwarz [36,37,38]

**Table 2:** Orthodontic evaluation: children with RS versus healthy control group.

Measurement	Children with RSs	Healthy Controls	Difference	Chi^2^	Prob > Chi²
	%	%	Healthy—RS		
Mentalis muscle activity	[*n* = 17]	[*n* = 19]		3.18	0.07
Normal balanced	64.7	89.5	24.8		
Hyperactive	35.3	10.5	−24.8		
Lip position	[*n* = 14]	[*n* = 19]		2.89	0.09
Competent	85.7	100.0	14.3		
Incompetent	14.3	00.0	−14.3		
Posture	[*n* = 14]	[*n* = 19]		0.06	0.80
Physiological	13.3	10.5	−2.8		
Hypotonic	86.7	89.5	2.8		
Habits	[*n* = 13]	[*n* = 19]		0.88	0.35
Yes	30.8	52.6	21.9		
No	69.2	47.4	−21.9		
Stage of dentition	[*n* = 15]	[*n* = 19]		2.41	0.49
Mixed early	26.7	21.0	−5.6		
Rest period	13.3	36.8	23.5		
Mixed late	53.3	36.8	−16.5		
Early permanent	06.7	05.3	−1.4		
Angle Occlusion left-side	[*n* = 15]	[*n* = 20]		1.52	0.68
Class I	28.6	45.0	16.4		
Class II	57.1	50.0	7.1		
Class III	14.3	5.0	−9.3		
Angle Occlusion right-side	[*n* = 15]	[*n* = 20]		0.22	0.97
Class I	38.5	40.0	1.5		
Class II	53.8	55.0	1.2		
Class III	7.7	5.0	−2.7		
Midline shift	[*n* = 17]	[*n* = 20]			
Upper—dental				0.08	0.77
Matching to the midline	40.5	45.9	5.4		
Deviation < 5mm	5.4	8.1	2.7		
Deviation > 5mm	0.0	0.0	0.0		
Lower—dental				1.35	0.51
Coincident	18.9	27.0	8.1		
Deviation < 5mm	24.2	27.0	2.8		
Deviation > 5mm	2.7	0.0	−2.7		
Lower—mandibular				0.11	0.74
Coincident	85.7	89.5	3.7		
Deviation < 5mm	14.3	10.5	−3.8		
Deviation > 5mm	0.00	0.0	0.0		
Phoniation	[*n* = 14]	[*n* = 19]		2.20	0.33
Normal	85.7	84.2	−1.5		
Interdental	14.3	05.3	61.0		
Nasal	00.0	10.5	−10.5		
Airway exam tonsils	[*n* = 14]	[*n* = 19]		1.44	0.49
Normal	60.0	57.9	−2.1		
Small	6.7	0.0	−6.7		
Enlarged	33.3	42.1	8.8		
Swallowing patterns	[*n* = 14]	[*n* = 19]		0.03	0.85
Somatic	71.4	68.4	−3.0		
Visceral	28.6	31.6	3.0		
Tongue position	[*n* = 14]	[*n* = 19]		0.11	0.75
Regular	78.6	73.7	−4.9		
Deep	21.4	26.32	4.9		
TMJ Check	[*n* = 19]	[*n* = 19]		7.13	<0.01 *
Conspicuous	31.6	00.0	−31.6		
Inconspicuous	68.4	100.0	31.6		
Breathing	[*n* = 15]	[*n* = 19]		5.99	0.05
Nose breathing	60.0	73.7			
Mouth breathing	13.3	26.3			
Snoring	26.7	0.0			
	**Median** **(range)**	**Median** **(range)**	***p*** **-value**		
Overjet (mm)	4 (2–10) [*n* = 17]	3 (0–9) [*n* = 20]	0.01 *		
Overbite (mm)	4.25 (2–6) [*n* = 16]	3 (−3–6) [*n* = 19]	0.07		
Mouth opening distance incisors (mm)	36 (28–51) [*n* = 16]	44 (36–55) [*n* = 12]	0.01 *		
Jaw Index	4.15 (1.87–9.55) [*n* = 16]	2.98 (0–8.96) [*n* = 20]	0.02 *		

Abbreviations: RS = Robin sequence; TMJ = Temporomandibular joint; SD = Standard deviation. * Significance level < 0.05.

**Table 3:** Values of facial soft tissue analysis of children with Robin sequence (RS) compared to the control group [36,37,38].

Measurement	Children with RS [*n* = 21]	Healthy Controls [*n* = 22]	*p*-Value
	Median (range)	Median (range)	
Profile angle (SnPog’-Pn [10°])	23 (14–29)	11.00 (9–23)	<0.001 *
Facial convexity angle (Gl’-Sn-Pog’ [167.0 ± 5.4°])	157 (149–173)	159.00 (149–170)	0.01 *
Merrifield z-angle (71.8°)	62.1 (42–82.3)	71.30 (60.4–84.4)	<0.001 *
Nasolabialangle (Cotg-Sn-Ls [106.5°])	112.6 (100–132.3)	114.5 (102.7–138.4)	0.90
Angle of the lowerface (Sn-Tra-Me’ [44.8 ± 3.1°])	39.6 (28.8–48.9)	35.8 (31.7–39.5)	0.01 *
Angle of the midface (N’-Tra-Sn [28.2 ± 2.6°])	27.1 (21.5–31.5)	25.5 (19.6–30)	0.01 *
Relation between higher/lower face (Tri-NR/NL:NR/NL-f-Me [161.80%])	157.5 147.1–211.8)	168.3 (147.3–193.2)	0.26
Relation between mid-/lowerface (f-Gl-Sub: Sub-f-Me [100%])	100 (84–147)	100 (90–110)	0.86
Relation between length and width of the face (TH-F-Me: WR-WL [161.80%])	155.5 (145.8–175.9)	162.55 (146.4–182.5)	0.14
Chin angle (GoL-f-Me-GoR [66%])	111 (98–131)	111 (97–124)	0.97

Abbreviation: RS = Robin sequence. * Significance level < 0.05.

**Table 4:** Digital study casts analysis of children with Robin sequence (RS) versus healthy control group. The total mesio-distal width of the four incisors of the upper and lower jaws according to Moyers and the anterior/posterior arch width measurements of the upper and lower jaws according to Harth were evaluated [39,40].

Measurement	Children with RS [*n* = 21]	Healthy Controls [*n* = 22]	*p*-Value
	Median (range)	Median (range)	
Moyers analysis			
Si LJ (mm)	22.9 (20.3–24.2)	23.2 (19.3–25.7)	0.32
SI UJ (mm)	30.00 (22.9–34)	30.2 (25.7–35.6)	0.85
Tonn Index UJ/LJ (%)	72.5 (69–80)	76 (66–90)	0.03 *
Discrepancy Z1Q (mm)	−0.35 (−6.3–2.4)	−0.2 (−20.8–3.9)	0.65
Discrepancy Z2Q (mm)	0 (−3.4–4.2)	0.2 (−2.4–2.3)	0.39
Discrepancy Z4Q (mm)	0.5 (−5.2–2.5)	0.6 (−7.5–3.4)	0.53
Discrepancy Z3Q (mm)	−0.95 (−11–3.5)	0.55 (−0.8–2.31)	0.19
Harth analysis			
AAWUJ (mm)	36 (32–40)	36 (32–40)	0.53
AAWLJ (mm)	35 (31–39)	36 (31–47)	0.57
PAWUJ (mm)	48 (42–55)	46 (35–51)	0.01 *
PAWLJ (mm)	48 (36–55)	49 (42–56)	0.36

Abbreviation: RS = Robin sequence; SD = Standard deviation; LJ = Lower jaw; UJ = Upper jaw; Si LJ = Total mesio-distal width of the four incisors of the lower jaw; SI UJ = Total mesio-distal width of the four incisors of the upper jaw; Z1Q = Zone between distal edge of lateral incisor and mesial edge of first molar in first quadrant; Z2Q = Zone between distal edge of lateral incisor and mesial edge of first molar in second quadrant; Z3Q = Zone between distal edge of lateral incisor and mesial edge of first molar in third quadrant; Z4Q = Zone between distal edge of lateral incisor and mesial edge of first molar in fourth quadrant; AAWUJ = Anterior arch width upper jaw; AAWLJ = Anterior arch width lower jaw; PAWUJ = Posterior arch width upper jaw; PAWLJ = Posterior arch width lower jaw. * Significance level < 0.05.

**Table 5:** Comparison of skeletal cephalometric measurements according to Hasund analysis [42], Posterior airway space (PAS) and soft tissue cephalometric analysis according to A.M. Schwarz [36,37] comparing children with Robin sequence (RS) against the healthy control group.

Measurement	Children with RS [*n* = 11]	Healthy Controls [*n* = 6]
	Median (range)	Median (range)
PAS (mm)		
Maxilla plane (20 mm)	12.5 (6.2–18.4)	17.05 (9.2–22.3)
Occlusal plane (9 mm)	8 (3–14)	7.5 (6–10)
Mandibular plane (13 mm)	11 (4–12)	9 (5–13)
**Hasund analysis**		
SNA (82 ± 3°)	77 (70.6–82.4)	80.5 (76.6–82.3)
SNB (80 ± 3°)	73.8 (63–80.7)	75.9 (67.3–79)
ANB (2.0 ± 2°)	4.8 (1–8.7)	4.9 (1.4–9.7)
Indiv. ANB	3.3 (1.4–7.4)	4.1 (0.9–6.8)
SN-Pg (82 ± 3°)	68.8 (62.4–77.9)	77.4 (66.6–79.8)
NS-Ba (130 ± 6°)	128.7 (113.3–135.38)	127.3 (122.1–139.2)
Ar-Go-Gn (126 ± 10°)	131.6 (118–142.4)	126.9 (120.3–143.8)
Holdaway angle (9.2°)	15.9 (5.8–24.7)	15.2 (6.7–24.1)
Interincisal angle 1-1 (131 ± 6°)	144.2 (125.2–161.7)	129.7 (116.4–140.7)
Nasolabial angle (109.8°)	103.1 (94–121.5)	106.7 (97.5–124.1)
OK1-NA (22.0 ± 3°)	15.8 (6.3–26.1)	19 (13.4–39.1)
UK1-NB (25.0 ± 3°)	15.2 (5.2–29.5)	23.6 (14.7–29.8)
OK1O-NA (4.0 ± 2 mm)	0.7 (−3.6–4)	1 (−2.1–4.6)
UK1O-NB (4.0 ± 2 mm)	0.6 (−1.6–7)	5.3 (0.3–6.8)
ML-NSL (32.0 ± 6°)	37.9 (20.9–69.1)	33.1 (25.3–48.7)
NL-NSL (8.5 ± 3°)	14.5 (6.1–22.1)	6.4 (4.2–19.7)
ML-NL (23.5 ± 3°)	23.9 (14.8–50.4)	26.5 (16.1–38)
Hasund Index (N-Sp’/Sp’-Gn [80.1%])	78.3 (69.9–84.8)	81.2 (65.9–89.3)
**Tübingen analysis**		
Wits appraisal (-0.3 ± 0.3 mm)	0.9 (−4.7–5.6)	2.6 (−0.6–4)
Mandibulary length (Go-Pog’ [75.7 mm])	69.9 (51.4–78.9)	76.9 (74.7–83.7)
Posterior mandibular body height (Go-Cond’ [54.2 mm])	51.3 (41.9–63.5)	52.2 (44.8–56)
Maxillary length (Spp-A’ [63.4 mm])	62.05 (51.7–70.4)	63.4 (61.7–69.2)
S-Go: N-Me (63,5 ± 1.5%)	62.1 (39.1–71.5)	64.8 (52.7–73.1)
NSpP: SpPMe (79.5%)	78 (70.1–83.7)	81.15 (67–88.4)

Abbreviation: RS = Robin sequence; PAS = Posterior airway space. Significance level < 0.05.

## Data Availability

Data is not available due to data protection of patients.
